# Fibronectin extra domain A (FN-EDA) elevates intraocular pressure through Toll-like receptor 4 signaling

**DOI:** 10.1038/s41598-020-66756-6

**Published:** 2020-06-17

**Authors:** Amanda L. Roberts, Timur A. Mavlyutov, Tanisha E. Perlmutter, Stacy M. Curry, Sherri L. Harris, Anil K. Chauhan, Colleen M. McDowell

**Affiliations:** 1 North Texas Eye Research Institute, Department of Pharmacology and Neuroscience, University of North Texas Health Science Center, Fort Worth, Texas United States; 2 Department of Internal Medicine, University of Iowa, Iowa City, IA United States; 3 Department of Ophthalmology and Visual Sciences, McPherson Eye Research Institute, University of Wisconsin-Madison, Madison, WI United States

**Keywords:** Animal disease models, Mechanisms of disease

## Abstract

Elevated intraocular pressure (IOP) is a major risk factor for the development and progression of primary open angle glaucoma and is due to trabecular meshwork (TM) damage, which leads to impaired aqueous humor outflow. Here, we explore a novel molecular mechanism involved in glaucomatous TM damage. We investigated the role of an endogenous Toll-like receptor 4 (TLR4) ligand, fibronectin-EDA (FN-EDA), in TGFβ2-induced ocular hypertension in mice. We utilized transgenic mouse strains that either constitutively express only FN containing the EDA isoform or contain an EDA-null allele and express only FN lacking EDA, with or without a mutation in *Tlr4*, in our inducible mouse model of ocular hypertension by injection of Ad5.TGFβ2. IOP was measured over time and eyes accessed by immunohistochemistry for total FN and FN-EDA expression. Constitutively active EDA caused elevated IOP starting at 14 weeks of age. Ad5.TGFβ2 induced ocular hypertension in wildtype C57BL/6J mice and further amplified the IOP in constitutively active EDA mice. TLR4 null and EDA null mice blocked Ad5.TGFβ-induced ocular hypertension. Total FN and FN-EDA isoform expression increased in response to Ad5.TGFβ2. These data suggest that both TLR4 and FN-EDA contribute to TGFβ2 induced ocular hypertension.

## Introduction

Glaucoma is a heterogeneous group of optic neuropathies with progressive degeneration of the optic nerve leading to vision loss and irreversible blindness^1^. The prevalence of individuals diagnosed with glaucoma worldwide by 2020 is predicted to be over 80 million and by 2040 over 112 million^2^. Glaucoma is characterized by cupping of the optic disc, death of retinal ganglion cells, and optic nerve degeneration^1^. Primary open angle glaucoma (POAG) is the most common form of glaucoma^3^ and elevated intraocular pressure (IOP) is the most significant causative risk factor for glaucoma^4^. IOP is the fluid pressure inside the eye that is regulated by the production of aqueous humor in the ciliary body and drainage of aqueous humor by the trabecular meshwork (TM) and uveoscleral outflow^5,6^. As a result of the aqueous humor primarily passing through the conventional TM outflow pathway, the TM is the major regulator of IOP. The TM is a biomechano-sensitive tissue located at the junction of the iris and cornea. The TM is composed of a series of fenestrated beams and sheets of extracellular matrix (ECM) covered with endothelial-like TM cells^7,8^. Of the three regions of the TM (uveal, corneosclearal, and cribiform), the cribiform region and the inner wall of the Schlemm’s canal, is the major site of aqueous humor outflow resistance^7^. Overall, the ECM composition of the TM is important in regulating aqueous humor outflow and forming a fluid flow pathway for proper aqueous humor drainage^9,10^. It is also known that POAG patients have an increased accumulation of ECM proteins within the TM^11,12^, increased ECM production leads to an increase in aqueous humor (AH) outflow resistance^13^, decreases AH outflow facility^14–18^, and causes ocular hypertension^13,15,19,20^.

Although the pathology of the disease is well studied, many of the current drug therapies used to lower elevated IOP focus on suppressing the aqueous humor formation and enhancing uveoscleral outflow; however, these particular therapies do not target the molecular pathology of the disease at the TM. Many of these therapies are also not uniformly effective, can progressively lose efficacy, and only slow vision loss progression^21^. Recently, new therapies have begun to target the TM and the underlying pathology such as the Rho kinase/norepinephrine transporter inhibitor netarsudil^22^. However, there is a still a need to identify additional novel molecular mechanisms responsible for glaucomatous damage in which a drug therapy can target the pathology of the disease to lower elevated IOP and prevent further TM damage.

The transforming growth factor beta 2 (TGFβ2) signaling pathway has been well studied in the TM and it is known to be elevated in the aqueous humor and TM tissue of glaucomatous eyes^23–26^. TGFβ2 has also been shown to mediate fibrosis development and ECM deposition within the TM, and induce ocular hypertension in both mice and in *ex vivo* perfusion organ culture systems^13,15,20,27,28^. We and others have also shown that TGFβ2 signals through both the canonical SMAD pathway, alters the ECM in human TM cells^29–35^, is essential for TGFβ2-induced ocular hypertension in mice^36^, and has the ability to crosstalk with TLR4 signaling^13,15^.

TGFβ2 signaling increases the production of ECM proteins, including fibronectin (FN). We and others have identified FN, a dimeric multidomain ECM glycoprotein, to be elevated in glaucomatous TM tissues and aqueous humor^15,18,28,37^. FN functions as a regulator of cellular processes, directs and maintains tissue organization and ECM composition, directs ECM-ECM and ECM-cell interactions, and regulates activity of growth factors and proteins associated with ECM remodeling. The multi-domain dimer is composed of type I, type II, and type III domains with over 20 alternatively spliced isoforms. FN is composed of either cellular FN (cFN) or plasma FN (pFN) isoforms. cFN has multiple isoforms generated by alternative processing of a single primary transcript at 3 domains: extra domain A (EDA), extra domain B (EDB), and the type III homologies connecting segment^38^. During embryonic development, the fibronectin EDA (FN-EDA) isoform is abundant^39^; however, in adults the presence of FN-EDA is minimal and primarily functions as a structural scaffold and signaling molecule that regulates cell adhesion, proliferation, and migration^40^. In addition, the expression of FN-EDA is upregulated as a response to tissue injury, repair, or remodeling^41^, and during disease states such as epithelial fibrosis^42^, wound healing^43^, and rheumatoid arthritis^44^. Importantly, FN-EDA isoform is elevated in glaucomatous trabecular meshwork tissue compared to normal trabecular meshwork tissue^12^ and amplifies the response of TGFβ2 in primary TM cells in culture^15^.

Recently, we discovered that FN-EDA enhances the TGFβ2-induced ECM response in primary TM cells, and this effect can be blocked by inhibition of toll-like receptor 4 (TLR4) signaling^15^. TLR4 is a member of the TLR family of proteins. Historically, TLR4 was first identified as the receptor for lipopolysaccharide (LPS). It is now known that TLR4 can also be activated by damage associated molecular patterns (DAMPs) as a result of tissue damage, cell injury, or ECM remodeling in other diseases^45–47^. FN-EDA is a known DAMP and activator of TLR4^48^. Our data suggests that TGFβ2 signaling increases ECM production, including production of FN-EDA, leading to activation of TLR4, and increased IOP. Activation of TLR4 downregulates the TGFβ2 antagonist, BMP and activin membrane bound inhibitor (BAMBI), leading to uninhibited TGFβ2 signaling, and a continuation of a pathogenic feed forward loop^45^. These data suggest a TGFβ2-TLR4 signaling crosstalk in the development of glaucomatous TM damage. Here, we identify the importance of FN-EDA in the development of ocular hypertension using transgenic mice that either constitutively express the EDA isoform or contain an EDA null copy, with or without knockdown of *Tlr4*.

## Results

### Anterior segment anatomy of transgenic mice

**Figure 1:**
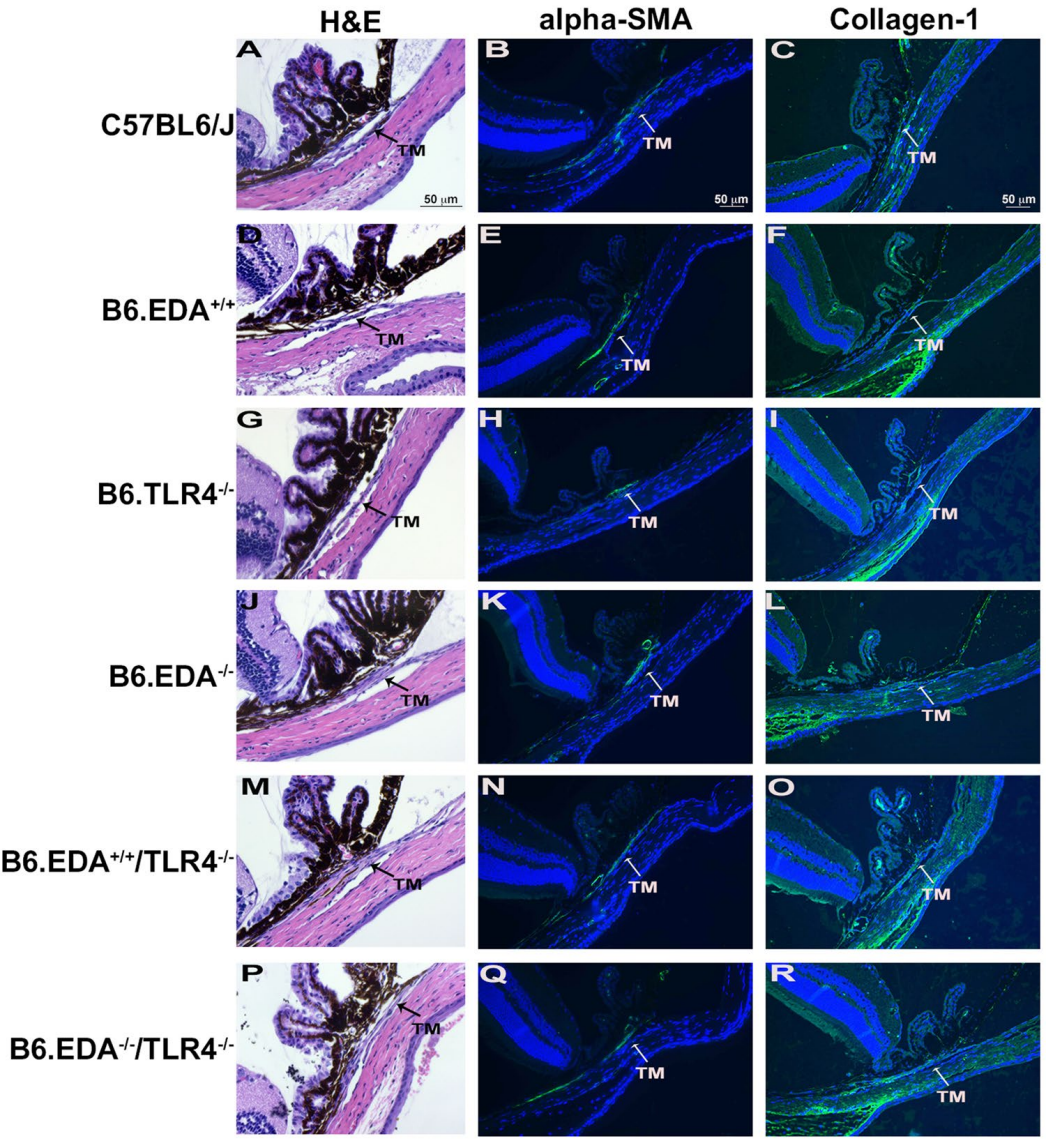
Histological exam of trabecular meshwork of EDA and TLR4 transgenic mice. (**A,D,G,J,M,P**) H&E staining of histological sections from C57BL/6J, B6.EDA^+/+^ B6.TL4^−/−^, B6.EDA^−/−^, B6.EDA^+/+^/TLR4^−/−^, and B6.EDA^−/−^ /TLR4^−/−^ mice. (**B, E, H, K, N, Q**) Alpha-SMA labeling of the TM in C57BL/6J, B6.EDA^+/+^ B6.TL4^−/−^, B6.EDA^−/−^, B6.EDA^+/+^/TLR4^−/−^, and B6.EDA^−/−^ /TLR4^−/−^ mice. (**C,F,I,L,O,R**) Collagen-1 labeling of the TM in C57BL/6J, B6.EDA^+/+^ B6.TL4^−/−^, B6.EDA^−/−^, B6.EDA^+/+^/TLR4^−/−^, and B6.EDA^−/−^ /TLR4^−/−^ mice. All animals were 60 days old at time of analysis, n = 5 mice/strain. All images taken at 200x magnification.

Previously, Chauhan and colleagues generated and characterized several EDA and TLR4 transgenic mouse strains used here: B6.EDA^−/−^ (EDA null), B6.TL4^−/−^ (TLR4 null), B6.EDA^+/+^ (constitutively active EDA), B6.EDA^−/−^/TLR4^−/−^, and B6.EDA^+/+^/TLR4^−/−^^49,50^. To determine whether there are any gross anatomical changes to the eye of these transgenic mice, we performed a clinical slit-lamp exam comparing each strain to C57BL/6J controls. Both frontal and lateral images were taken of the exterior eye globe at 15, 30, and 60 days post-natal (Supplemental Fig. 1). Irises of all mouse strains at each age group appear densely pigmented with a complex morphology, as previously described for wildtype C57BL/6J mice^50^. The pupils are round, corneas clear, and no obvious clinically observed anterior segment morphometric abnormalities. In addition, histological sections and H&E analysis was performed on the same mice at each time point (Supplemental Fig. 2). The iridocorneal angles are open and TM morphology appears normal for all strains and ages analyzed. Our data suggests that there are no anatomical differences in the anterior segment of the mouse eyes between strains and there no obvious developmental morphometric abnormalities in B6.EDA^−/−^, B6.TL4^−/−^, B6.EDA^+/+^, B6.EDA^−/−^ /TLR4^−/−^, or B6.EDA^+/+^/TLR4^−/−^ mice. We also characterized the TM of each mouse strain at 60 days of age (Fig. 1). All mouse strains showed a defined TM and Schlemm’s canal by H&E staining. The TM of all mouse strains stained positive for alpha-SMA, a known protein highly expressed in TM cells, and collagen-1, a known ECM marker in the TM. These data suggest that the TM in all the mouse strains analyzed is present and anatomically normal.

### Constitutively active FN-EDA causes elevated IOP in mice

**Figure 2:**
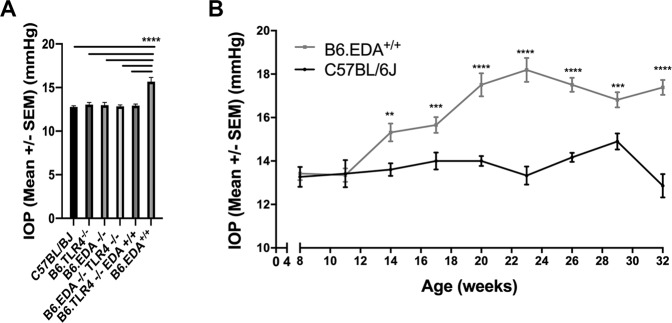
Constitutively active FN-EDA causes elevated IOP in mice: **(A)** IOP at 5 months of age in C57BL/6J (n = 17), B6.TL4^−/−^ (n = 8), B6.EDA^−/−^ (n = 18), B6.EDA^−/−^ /TLR4^−/−^ (n = 23), B6.EDA^+/+^/TLR4^−/−^ (n = 16), and B6.EDA^+/+^ (n = 8) mice. B6.EDA^+/+^ mice had significantly elevated IOP compared to each of the other strains. Statistical significance determined by one-way ANOVA followed by Tukey analysis. **(B)** IOP from 8 weeks of age to 32 weeks of age in C57BL/6J and B6.EDA^+/+^ mice. There was no significant difference in IOP between C57BL/6J and B6.EDA^+/+^ mice at 8–12 weeks of age. At 14 weeks of age, ocular hypertension developed in B6.EDA^+/+^ mice and remained elevated through 32 weeks of age compared to C57BL/6J mice. Statistical significance determined by Student’s t-test at each time point. At least n = 6–20 eyes (3–10 mice) were measured at each time point. ****P < 0.0001, ***P < 0.001, **P < 0.05.

Clinical and histological analysis of the transgenic EDA and TLR4 strains demonstrated normal TM development. However, when comparing baseline IOP readings from each mouse strain (C57BL/6J, B6.EDA^−/−^, B6.EDA^−/−^ /TLR4^−/−^, B6.EDA^+/+^/TLR4^−/−^, B6.TL4^−/−^, and B6.EDA^+/+^) at 5 months of age, IOP in B6.EDA^+/+^ is significantly elevated compared to each of the other strains, including B6.EDA^+/+^/TLR4^−/−^ (Fig. 2A). These data suggest that EDA can cause elevated IOP and it is TLR4 dependent. To further analyze the effect of EDA on IOP, we measured IOP in C57BL/6J and B6.EDA^+/+^ mice starting at age 8 weeks through 32 weeks of age (Fig. 2B). Significant IOP elevation began at 14 weeks of age in B6.EDA^+/+^ mice compared to C57BL/6J controls and continued through 32 weeks of age. As expected, C57BL/6J mice maintained a normal IOP throughout the 32 weeks as previously reported^15^. These data demonstrate that although the TM develops normally in EDA^+/+^ mice, and EDA^+/+^ mice at 8 weeks of age have a normal IOP, constitutively active EDA causes elevated IOP to develop over time.

### FN-EDA and TLR4 are necessary for TGFβ2 induced ocular hypertension

**Figure 3:**
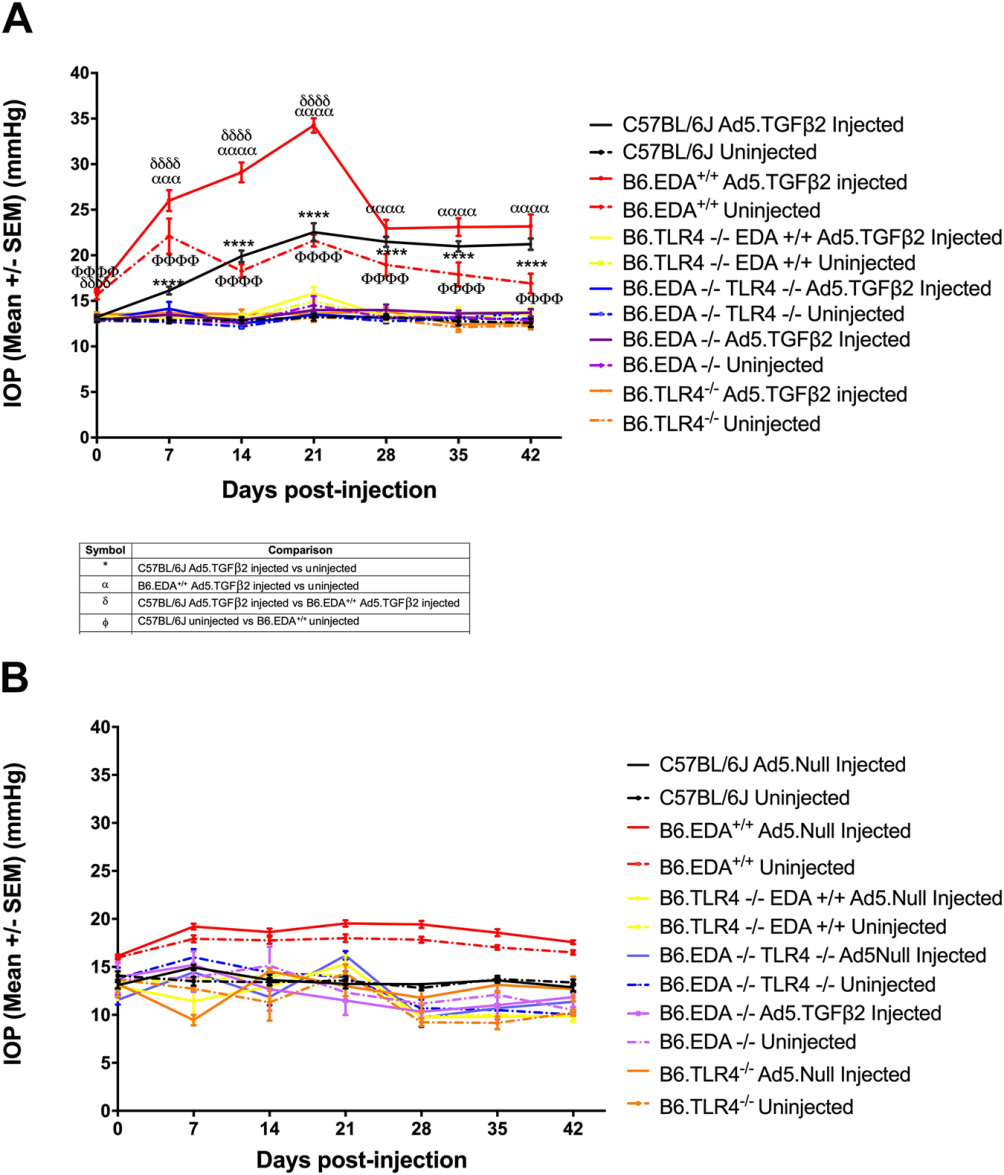
FN-EDA and TLR4 are necessary for TGFβ2-induced ocular hypertension: Ad5.TGFβ2 significantly elevated IOP in C57BL/6J mice (16.1 +/− 0.4 mmHg) and B6.EDA^+/+^ mice (26.0 +/− 1.1 mmHg) compared to their respective uninjected contralateral eyes (C57BL/6J: 12.8 +/− 0.3 mmHg; B6.EDA^+/+^: 22.1 +/− 1.9 mmHg) starting at 7-days post injection and remained significant through 42 days post-injection (C57BL/6J injected: 22.2 +/− 0.6 mmHg, C57BL/6J uninjected: 12.6 +/− 0.5 mmHg; B6.EDA^+/+^ injected: 23.2 +/− 1.3 mmHg, B6.EDA^+/+^ uninjected 16.9 +/− 1.1 mmHg). Ad5.TGFβ2 elevated IOP in B6.EDA^+/+^ mice significantly above the IOP in Ad5.TGFβ2 injected C57BL/6J mice through 21 days post-injection (C57BL/6J injected: 22.5 +/− 1.0 mmHg; B6.EDA^+/+^ injected: 34.3 +/− 0.8 mmHg). C57BL/6J uninjected eyes compared to B6.EDA^+/+^ uninjected eyes were significantly different throughout the time course. Ad5.TGFβ2 had no effect on B6.TLR4^−/−^, B6.EDA^−/−^, B6.EDA^+/+^/TLR4^−/−^, or B6.EDA^−/−^ /TLR4^−/−^ mice at any time point. Average IOP at day 42 for B6.TLR4^−/−^ 12.6 +/− 0.3 mmHg injected, 12.3 +/− 0.4 mmHg uninjected; B6.EDA^−/−^ 13.7 +/− 0.4 mmHg injected, 12.9 +/− 0.2 mmHg uninjected; B6.EDA^+/+^/TLR4^−/−^ 13.8 +/− 0.5 mmHg injected, 13.5 +/− 0.3 mmHg uninjected; B6.EDA^−/−^/TLR4^−/−^ 13.7 +/− 0.3 mmHg injected, 13.1 +/− 0.2 mmHg uninjected. C57BL/6J (*n* = 17), B6.EDA^−/−^ (n = 18), B6.EDA^+/+^ mice (n = 8), B6.TLR4^−/−^ mice (n = 8), B6.EDA^−/−^ /TLR4^−/−^ (n = 21–23) and B6.EDA^+/+^/ TLR4^−/−^ (n = 16). Statistical significance determined by one-way ANOVA followed by Tukey analysis at each time point, *, α, δ, ϕ, ω all indicate significance of at least P < 0.001. All values represent mean +/− SEM. **(B)** Ad5.Null had no effect on IOP in C57BL/6J, B6.EDA^+/+^ B6.TL4^−/−,^ B6.EDA^−/−^, B6.EDA^+/+^/TLR4^−/−,^ or B6.EDA^−/−^ /TLR4^−/−^ mice compared to the contralateral uninjected eye at any time point. N = 3-5 mice/strain.

To test the crosstalk between TGFβ2 and TLR4, we utilized our established mouse model of ocular hypertension using Ad5.TGFβ2 virus, which contains a bioactivated form of TGFβ2^15,19,36,51^. Ad5.TGFβ2 was injected intravitreally into one eye of each animal and the contralateral uninjected eye was used as a negative control. In order to determine whether FN-EDA and TLR4 are necessary for Ad5.TGFβ2-induced ocular hypertension phenotype, we tested wildtype C57BL/6J (*n* = 17), B6.TLR4^−/−^ mice (n = 8), B6.EDA^+/+^ mice (8), B6.EDA^−/−^ (n = 18), B6.EDA^−/−^ /TLR4^−/−^ (n = 23) and B6.EDA^+/+^/ TLR4^−/−^ (n = 16) mice in our model system (Fig. 3A). As expected, C57BL/6J mice eyes injected with Ad5.TGFβ2 developed ocular hypertension compared to their contralateral uninjected eye^15,51^. Ad5.TGFβ2 also significantly elevated IOP in B6.EDA^+/+^ mice compared to their contralateral uninjected eye from days 7-42 post-injection. In addition, the IOP in Ad5.TGFβ2 injected eyes from B6.EDA^+/+^ mice are significantly elevated compared to Ad5.TGFβ2 injected eyes in wildtype C57BL/6J mice from days 0-21 post-injection, demonstrating an enhanced effect in these mice. Similar to the data in Fig. 2, uninjected B6.EDA^+/+^ eyes have significant IOP elevation compared to uninjected C57BL/6J eyes. Previously, we reported that C3H/HeJ mice harboring a spontaneous mutation in *Tlr4* are also resistant to TGFβ2-induced ocular hypertension^15^. Here, we recapitulate this data on the C57BL/6 J (B6) genetic background in B6.TLR4^−/−^ mice which had no significant IOP changes in response to Ad5.TGFβ2 injection, and in B6.EDA^+/+^/TLR4^−/−^ mice as mutation in *Tlr4* blocked EDA and TGFβ2-induced IOP elevation. Mutation in EDA^−/−^ also blocked TGFβ2-induced ocular hypertension compared with C57BL/6J controls and uninjected control eyes. In addition, similar to our previous reports Ad5.Null virus has no effect on IOP in any mouse strain (Fig. 3B)^15^. These data suggest that FN-EDA and TLR4 are both necessary for TGFβ2-induced ocular hypertension.

### TGFβ2 increases FN and FN-EDA expression in the trabecular meshwork

**Figure 4:**
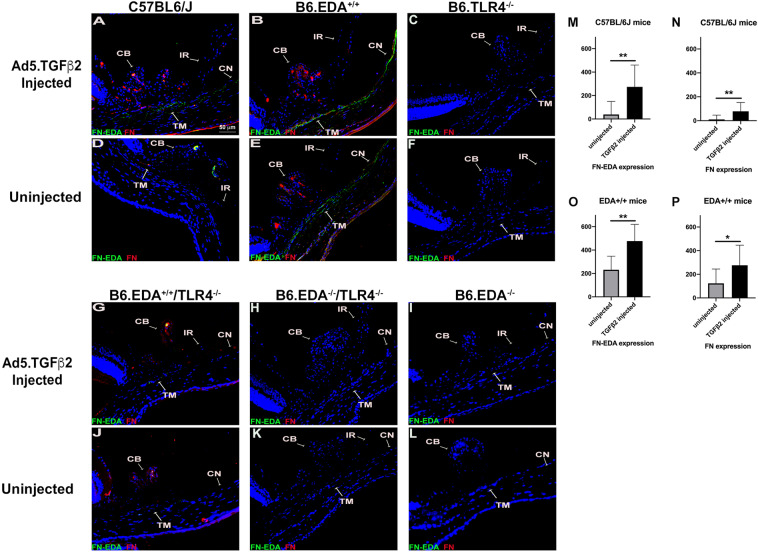
TGFβ2 induces FN and FN-EDA expression in C57BL/6J and EDA + / + mice. All analyses were performed 6-7 weeks post-injection of Ad5.TGFβ2. **(A,D)** Ad5.TGFβ2 increased both FN and FN-EDA expression in the TM of C57BL/6J mice. **(B,E)** Ad5.TGFβ2 increased both FN and FN-EDA expression in the TM of B6.EDA^+/+^ mice. **(C,F)** Ad5.TGFβ2 had no effect on FN or FN-EDA expression in the TM of B6.TLR4^−/−^ mice. **(G,J)** Trace amounts of FN-EDA and FN was detected in B6.EDA^+/+^/TLR4^−/−^ mice, no difference was observed between Ad5.TGFβ2 injected and uninjected eyes. **(H,I,K,L)** No detectable FN-EDA expression was observed in B6.EDA^−/−^ or B6.EDA^−/−^ /TLR4^−/−^ mice, and no difference was observed between Ad5.TGFβ2 injected and uninjected eyes. **(M,N)** FN and FN-EDA expression quantified by ImageJ analysis for mean intensity/area in the TM of C57BL/6J mice. **(O,P)** FN and FN-EDA expression quantified by ImageJ analysis for mean intensity/area in the TM of B6.EDA^+/+^ mice. C57BL/6J mice (n = 11), B6.EDA^+/+^ mice (n = 6), B6.TLR4^−/−^ mice (n = 8), B6.EDA^−/−^ (n = 18), B6.EDA^−/−^ /TLR4^−/−^ (n = 22) and B6.EDA^+/+^/ TLR4^−/−^ (n = 16). Statistical significance determined by Student’s paired t-test *P < 0.05.

Next, we explored the effect of TGFβ2 on FN and FN-EDA expression in the TM of each of the mouse strains (Fig. 4, Supplemental Figs. 3 and 4). Ad5.TGFβ2 significantly increased both FN levels and FN-EDA levels in the TM of C57BL/6J mice (Fig. 4A,D,M,N). B6.EDA^+/+^ mice also expressed the FN-EDA isoform in the TM of uninjected naïve eyes, and TGFβ2 further increased the expression of FN and EDA (Fig. 4B,E,O,P). TGFβ2 had no effect on FN or FN-EDA expression in the TM of B6.TLR4^−/−^ mice, demonstrating again that TLR4 is necessary for TGFβ2-induced TM damage (Fig. 4C,F). Trace amounts of FN and FN-EDA were evident in the TM of B6.EDA^+/+^/ TLR4^−/−^ mice (Fig. 4G,J), but there was no difference between TGFβ2-injected and control eyes. Correlating with the IOP data, TGFβ2 also had no effect on the expression of FN or FN-EDA in the TM of Ad5.TGFβ2-injected and uninjected eyes of B6.EDA^−/−^, B6.EDA^−/−^ /TLR4^−/−^, and B6.TL4^−/−^ mice (Fig. 4C,F,H,K,I,L).

## Discussion

Here we identified FN-EDA as a necessary molecule in pathogenic TGFβ2-TLR4 signaling cross-talk in the development of ocular hypertension in mice. Previously we showed that FN containing the EDA isoform can increase ECM production and enhance the effects of TGFβ2 in primary human TM cells in culture, and this effect could be blocked by inhibition of TLR4^15^. We also demonstrated that mutation in *Tlr4* blocked TGFβ2-induced ocular hypertension in mice^15^. We further expanded on this hypothesis here and demonstrated that exclusion of the EDA exon in B6.EDA^−/−^ mice blocked TGFβ2 induced ocular hypertension and constitutive inclusion of the EDA exon in B6.EDA +/+ caused ocular hypertension and further exacerbated the effect of TGFβ2. Importantly, the effect of EDA^+/+^ on ocular hypertension was dependent on TLR4. These data highlight a novel role of EDA in ocular hypertension and provides a new therapeutic target to lower IOP that is relevant to the pathology of glaucomatous TM damage.

TLR4 is a member of the TLR family of the innate immune system. Recent evidence suggests that endogenous ligands of cell compartments and matrix can activate TLR4, a phenomenon that can occur as a result of tissue damage or extracellular matrix remodeling^45–47^. These ligands, also known as damage associated molecular patterns (DAMPs), have the potential to cause a cellular response. Endogenous DAMPs include FN-EDA, HMGB-1, and low molecular weight hyaluronic acid amongst others. Here we recapitulate our earlier report that TLR4 is necessary for TGFβ2-induced ocular hypertension^15^, and further demonstrate FN-EDA is necessary and dependent on TLR4 for TGFβ2-induced ocular hypertension.

TLR4 is a relevant pathway to study in the context of glaucoma. Several polymorphisms have been identified in *TLR4* in human SNP studies of glaucoma patients. Shibuya el al identified multiple SNPs (rs10759930, rs1927914, rs1927911, rs12377632, rs2149356, and rs7037117) in the *TLR4* gene associated with the risk of normal tension glaucoma (NTG)^52^. In addition, Takano el al identified in Japanese individuals with POAG, NTG, and exfoliation glaucoma (XFG), the allele frequency of rs2149356 was the most significant. Further, the SNPs at rs10759930, rs1927914, rs1927911, and rs2149356 were all significantly higher in the glaucoma groups compared to the control group^53^. Navarro-Partida el al evaluated SNPs Asp299Gly (rs4986790 A/G) and Thr399lle (rs4986791 C/T) in Mexican patients with POAG compared to controls and found that the *TLR4* coding SNPs Asp299Gly and Thr399lle was significantly higher in the POAG patients, suggesting that there is a genetic susceptibility alleles for POAG in the Mexican population^54^. However, these results may be population specific as another report of a NTG Korean population demonstrated no statistical significance difference between the NTG patients and controls for SNPs (rs10759930, rs1927914, rs1927911, rs12377632, rs2149356, rs11536889, rs7037117, and rs7045953) in the *TLR4* gene^55^. And, Abu-Amero and colleagues evaluated the SNP at s4986790 in the *TLR4* gene of Saudi POAG patients, and found no statistical difference compared to controls^56^. Importantly, the role of TLR4 in fibrogenesis has also been identified and confirmed by specific SNP alleles in *TLR4* being associated with a delayed progression of fibrosis in liver disease and conferring an overall protective effect^57,58^.

Fibronectin is an extracellular glycoprotein that is elevated in the aqueous humor and glaucomatous TM tissues^18,37,38^. Fibronectin provides structural support, signaling and regulates growth factors involved in ECM remodeling. Interestingly, fibronectin can bind to itself, other ECM molecules, growth factors, and receptors. Cellular FN containing the EDA domain has been shown to play important roles in tissue damage^44,59–61^ and fibrogenesis^41,62–65^. Medina et al. showed that human normal TM and glaucomatous TM cells and tissues express the cFN isoforms (EDA and EDB) and that the EDA isoform is elevated in the glaucomatous TM tissue^66^. EDA expression is also induced by TGFβ2 and dexamethasone in primary TM cultures^12,67^. Here we demonstrate elevated expression of EDA in the mouse TM in response to TGFβ2.

Functionally, it is known that EDA acts as an endogenous ligand for toll-like receptor 4 (TLR4)^48^. The activation of TLR4 is also dependent upon the expression of MD-2 and other TLR4 accessory proteins^48,68^. Recently, α4β1 integrin was identified to function as a TLR4-coreceptor to initiate an EDA-dependent response^69^. In addition, increased EDA levels led to further production of the EDA isoform in dermal fibroblasts^69^. The mechanism in which FN-EDA activates TLR4 in TM cells remains to be identified, but these data support our hypothesis of a progressive feed-forward mechanism of pathogenic TLR4 signaling involving the fibronectin EDA isoform.

EDA null and constitutively active EDA mice with and without mutation of *Tlr4* were previously developed and characterized^41,49^. These transgenic mice provide a means to study the function of the EDA isoform and its dependence on Tlr4 in a controlled strain specific manner using our inducible model of ocular hypertension. Interestingly the TM of B6.EDA^−/−^ mice appears to develop normally as shown by gross clinical and histological analysis, and these mice are completely resistant to TGFβ2 induced ocular hypertension. B6.EDA^−/−^ mice also express very little FN in the TM. Previously, it has been shown that depletion of the EDA-domain can lead to a 40% decrease in the remaining FN levels^70^. However, this phenomenon appears to be tissue specific as it is also known that B6.EDA^−/−^ mice have normal levels of FN in the heart and brain^41^. These data highlight that the EDA isoform of FN is necessary for TGFβ2-induced ocular hypertension. Our data also demonstrates that TGFβ2 overexpression in the TM induces expression of both FN as well as expression of the EDA isoform in wildtype C57BL/6J mice and constitutively active EDA^+/+^ mice as measured 7-weeks after TGFβ2 injections, complimenting the increase in IOP in these mice. It is important to note that the anti–cFN-EDA antibody (NBP1-91258; Novus Biologicals) used here is made against the C-terminal region of the Fibronectin protein (within residues 2250–2300). Mouse-anti-FN antibody (clone IST-4; Sigma-Aldrich) recognizes an epitope located within the 5th type III repeat of human plasma fibronectin, which is common to all fibronectin forms. However, the exposure and accessibility of the 5th type III repeat can be affected by unfolding of the FN protein, FN-FN interactions during fibril formation, and placement or interaction within the ECM^71–73^. Therefore, the binding of this antibody may be affected in certain tissues and disease states and the presence or absence of the EDA domain. This hypothesis is supported by previous studies where both antibodies were used in human TM tissues, and a similar phenotype was noted as we see here, where EDA did not always co-localize with the total FN antibody^12^. Therefore, it is likely that we are underestimating the amount of total FN produced in our experiments.

We also demonstrated that when EDA is constitutively expressed the mice develop high IOP by 14 weeks of age and persists for at least 32 weeks of age. Therefore, constitutively active EDA mice represent a novel mouse model of ocular hypertension. These mice are an excellent resource for the field of ocular hypertension research and TM pathology as they develop ocular hypertension by an early age, maintain open irideocorneal angles, and produce a homogenous response between animals. IOP was also amplified in the TGFβ2 injected eyes of B6.EDA^+/+^ mice compared to the TGFβ2 injected eyes of C57BL6/J mice for 21 days post-injection. Starting at 28 days post-injection the IOP in the TGFβ2 injected eyes of B6.EDA^+/+^ mice lowered to that of the C57BL/6J injected eyes. However, the IOP in the TGFβ2 injected eyes of B6.EDA^+/+^ mice remained significantly elevated compared to their uninjected control eye from day 14 until the end of the time course. These data suggest that there may be an additional compensatory mechanism that is regulating the IOP with the overexpression of both EDA and TGFβ2. We have previously shown a decrease in IOP at around 21 days after Ad5.TGFβ2 injection in other mouse strains^36^ which is thought to be due to genetic background differences. Therefore, it could be that the constitutive expression of EDA not only causes elevated IOP and an initial amplification of TGFβ2 responses, but it may also induce a compensatory pathway that can partially reduce the effects. Further analysis of EDA^+/+^ mice and downstream signaling pathways are needed to fully address this phenomenon. A more detailed analysis of the TM over time will help elucidate the exact molecular and pathological changes occurring in the TM that result in the ocular hypertension phenotype. In addition, further characterization will determine whether this TM damage leads to additional glaucomatous phenotypes in the retina ganglion cells and optic nerve of these animals.

In conclusion, we have demonstrated that both Tlr4 and FN-EDA are necessary for TGFβ2-induced ocular hypertension. These data provide a model system to study glaucomatous TM damage and develop novel therapeutic strategies.

## Materials and Methods

### Animals and Adenovirus Injection

All experiments were conducted in compliance with the ARVO Statement for the Use of Animals in Ophthalmic and Vision Research and approved by the University of North Texas Health Science Center (UNTHSC; Fort Worth, TX, USA) and the University of Wisconsin-Madison (Madison, WI) Institutional Animal Care and Use Committee (IACUC) Guidelines and Regulations. The generation of FN-EDA^−/−^, FN-EDA^+/+^, FN-EDA^+/+^/TLR4^−/−^, and FN-EDA^−/−^/ TLR4^−/−^ mice has previously been described^41^. B6.EDA^+/+^ mice were generated to contain spliced sites at both splicing junctions of the EDA exon and therefore constitutively express only FN containing EDA. B6.EDA^−/−^ mice contain an EDA-null allele of the EDA exon and express only FN lacking EDA. All animals were housed in the UNTHSC vivarium or the UW-Madison vivarium. Adenovirus 5 (Ad5) viral vector expressing human TGFβ^c226s/c228s^ (referred to throughout as Ad5.TGFβ2) (University of Iowa, Iowa City, IA, USA) was used to overexpress TGFβ2 as previously described^34–36^. Ad5.Null virus (Vector Biolabs, Malvern, PA, USA) was used as a negative control. Briefly, 2 μL of 2.5 × 10^7^ plaque-forming units (pfu) was intravitreally injected into one eye, and the uninjected contralateral eyes were used as negative controls as previously described^13,15^.

### Intraocular Pressure Measurements

Intraocular pressure was measured as previously described^13^. Briefly, IOP was measured on isoflurane anesthetized mice using the Tonolab tonometer (Colonial Medical Supply, Franconia, NH, USA). All IOP measurements were performed during the same time period of the light-on phase. Statistical significance was determined by one-way ANOVA followed by Tukey analysis at each time point, comparing the injected eyes and contralateral uninjected eyes between each strain. All mice were at least 5 months old for adenoviral injection experiments.

### Immunohistochemistry of Mouse Eyes

After completion of the IOP time course after Ad5.TGFβ2 injection, mouse eyes were enucleated and fixed in 4% PFA overnight. Eyes were embedded in paraffin, cut into 5-μm sections, and transferred to glass slides. Slides were heated for 2 hours on a heated plate. Deparaffinization was performed by washing with xylene, 100% ethanol, and 95% ethanol, twice for 2 minutes each. Slides were soaked in distilled water and antigen retrieval (citrate buffer) was performed in 65 ^°^C water bath for 30 minutes. Tissues were cooled off to room temperature and washed with 1X PBS three times. Tissues were stained with hematoxylin and eosin or blocked using Superblock Blocking Buffer in PBS (Thermo Fisher Scientific) for 60 minutes and further processed for immunohistochemistry as follows. For TM morphology analysis, primary rabbit-anti-smooth muscle actin (1:00, ab5694, Abcam) and rabbit-anti-collagen-1 (1:100, ab34710, Abcam) were used followed by secondary antibody Alexa Fluor-labeled Donkey-anti Rabbit Ig (ThermoFisher), 1:500 dilution. For FN analysis, primary rabbit-anti–cFN-EDA antibody (1:100, NBP1-91258; Novus Biologicals) and mouse-anti-FN antibody (1:100, clone IST-4; Sigma-Aldrich) were used followed by secondary antibodies Alexa Fluor–labeled anti-rabbit Ig (Life Technologies) 1:500 dilution and Alexa Fluor-labeled donkey anti-mouse Ig (Life Technologies) 1:500. The mouse–anti-FN antibody recognizes epitopes on the N-terminus of FN, which are expressed by all FN isoforms. Slides were mounted with Prolong Gold mounting medium containing DAPI (Invitrogen-Molecular Probes). Images were taken using Keyence microscope BZ-X710 (Itasca, IL) or Zeiss Axio Imager Z2 microscope. All images were taken at ×200 magnification. Mean florescence intensity/area was measured in a masked manner for each image using ImageJ analysis. Statistical significance was determined by Student’s paired t-test for each animal comparing the TGFβ2-injected eye to the contralateral uninjected eye.

### Slit-lamp examination

Anterior segment phenotypes were assessed with a slit-lamp (SL-D7; Topcon, Tokyo, Japan) and photo documented with a digital camera (D100; Nikon, Tokyo, Japan). Images were taken with identical camera settings and prepared with identical image software processing.

## Supplementary information

Supplementary Information.

## Data Availability

All data generated or analyzed during this study are included in this published article.
