# Supplementary material for: Fibronectin extra domain A (FN-EDA) elevates intraocular pressure through Toll-like receptor 4 signaling
Source: Sci Rep. 2020 Jun 17;10:9815. doi: 10.1038/s41598-020-66756-6 (PMC7299944; doi:10.1038/s41598-020-66756-6)

Fibronectin extra domain A (FN-EDA) elevates intraocular pressure through Toll-like receptor 4 signaling

Amanda L. Roberts[1], Timur A. Mavlyutov[3], Tanisha E. Perlmutter[3], Stacy M. Curry[1], Sherri L. Harris[1], Anil K. Chauhan[2], Colleen M. McDowell[3]

[1]North Texas Eye Research Institute, Department of Pharmacology and Neuroscience, University of North Texas Health Science Center, Fort Worth, Texas, United States.

[2]Department of Internal Medicine, University of Iowa, Iowa City, IA, United States

[3]Department of Ophthalmology and Visual Sciences, McPherson Eye Research Institute, University of Wisconsin-Madison, Madison, WI, United States.

Corresponding author: Colleen M. McDowell, [2]Department of Ophthalmology and Visual Sciences, McPherson Eye Research Institute, University of Wisconsin-Madison, Madison, WI, United States. cmmcdowell@wisc.edu

Supplemental Figure 1. Clinical exam of anterior chambers of EDA and TLR4 transgenic mice. Clinical slit-lamp images of frontal and lateral views of anterior chamber in C57BL/6J, B6.EDA$^{-/-}$, B6.TL4$^{-/-}$, B6.EDA$^{+/+}$, B6.EDA$^{-/-}$ /TLR4$^{-/-}$, B6.EDA$^{+/+}$/TLR4$^{-/-}$ mice. No significant gross anatomical changes were identified 15, 30, and 60 days post-natal in any of the mouse strains.

Supplemental Figure 2. Histological exam of anterior chambers of EDA and TLR4 transgenic mice. H&E staining of histological sections from C57BL/6J, B6.EDA$^{-/-}$, B6.TL4$^{-/-}$, B6.EDA$^{+/+}$, B6.EDA$^{-/-}$ /TLR4$^{-/-}$, B6.EDA$^{+/+}$/TLR4$^{-/-}$ mice. No significant gross anatomical changes were identified 15, 30, and 60 days post-natal in any of the mouse strains. All images taken at 200x magnification.

Supplemental Figure 3. TGFβ2 induces FN and FN-EDA expression in C57BL/6J and EDA+/+ mice. (A-F) Ad5.TGFβ2 increased both total FN and FN-EDA expression in the TM of C57BL/6J mice. **(G-L)** Ad5.TGFβ2 increased total FN expression in the TM of B6.EDA$^{+/+}$ mice. **(M-R)** Ad5.TGFβ2 had no effect on FN or FN-EDA expression in the TM of B6.TLR4$^{-/-}$ mice. C57BL/6J mice (n=11), B6.EDA$^{+/+}$ mice (n=6), and B6.TLR4$^{-/-}$ mice (n=8).

Supplemental Figure 4. B6.EDA$^{-/-}$ and B6.TLR4$^{-/-}$ mice are resistant to TGFβ2-induced ECM changes in the TM. (A-F) Trace amounts of FN-EDA and FN was detected in B6.EDA$^{+/+}$/TLR4-/- mice, no difference was served between Ad5.TGFβ2

injected and uninjected eyes. **(G-R)** No detectable FN-EDA expression was observed in B6.EDA$^{-/-}$ or B6.EDA$^{-/-}$ /TLR4$^{-/-}$ mice, and no difference was observed between Ad5.TGFβ2 injected and uninjected eyes. B6.EDA$^{-/-}$ (n=18), B6.EDA$^{-/-}$ /TLR4$^{-/-}$ (n=22) and B6.EDA$^{+/+}$/ TLR4$^{-/-}$ (n=16).

Supplemental Figure 1

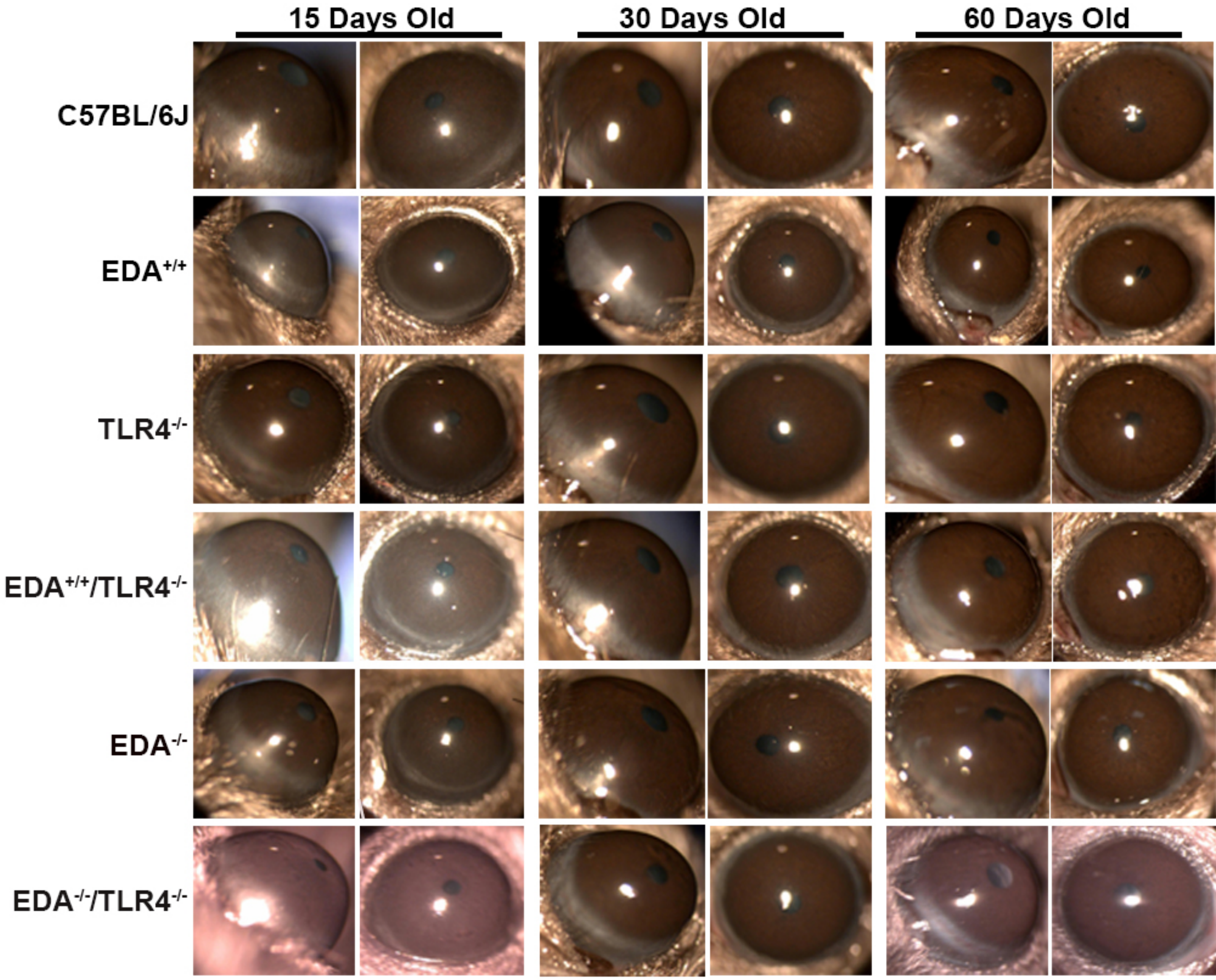

Supplemental Figure 2

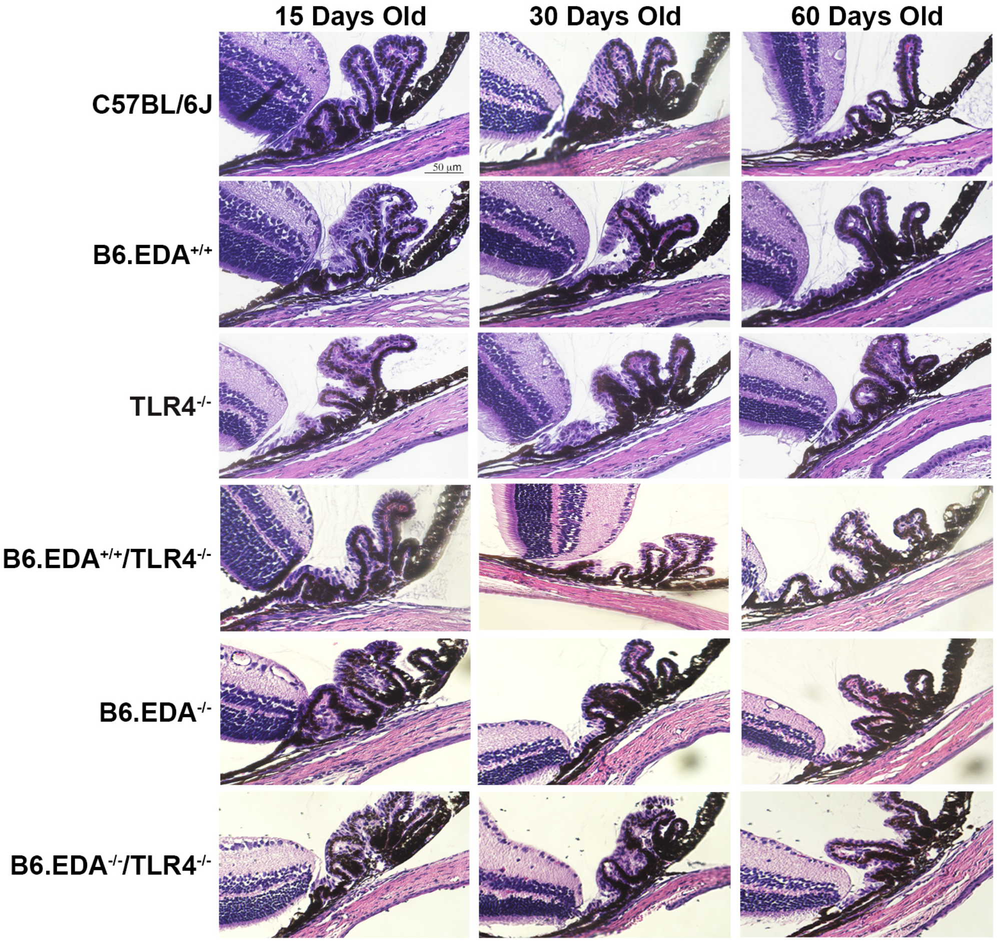

Supplemental Figure 3

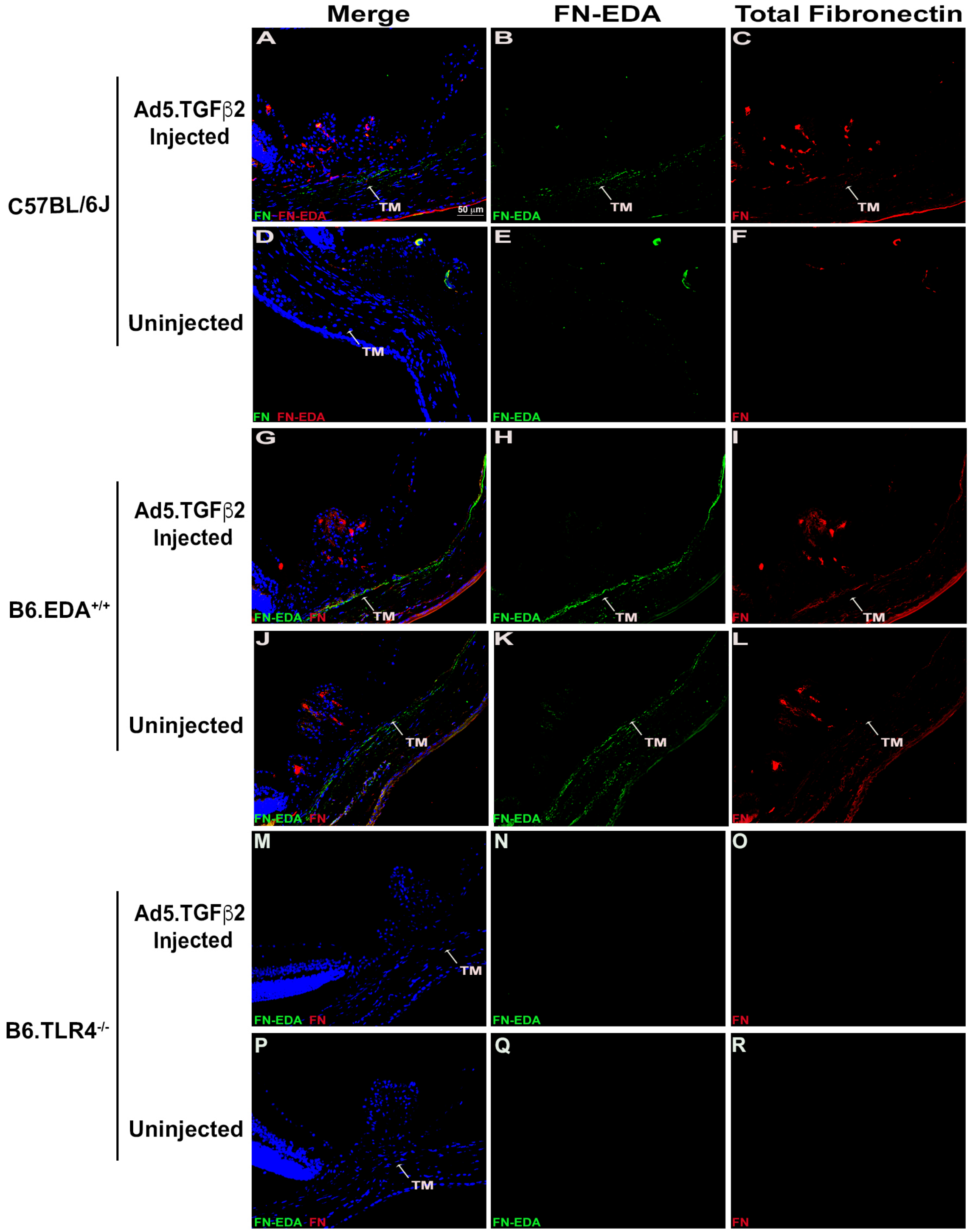

Supplemental Figure 4

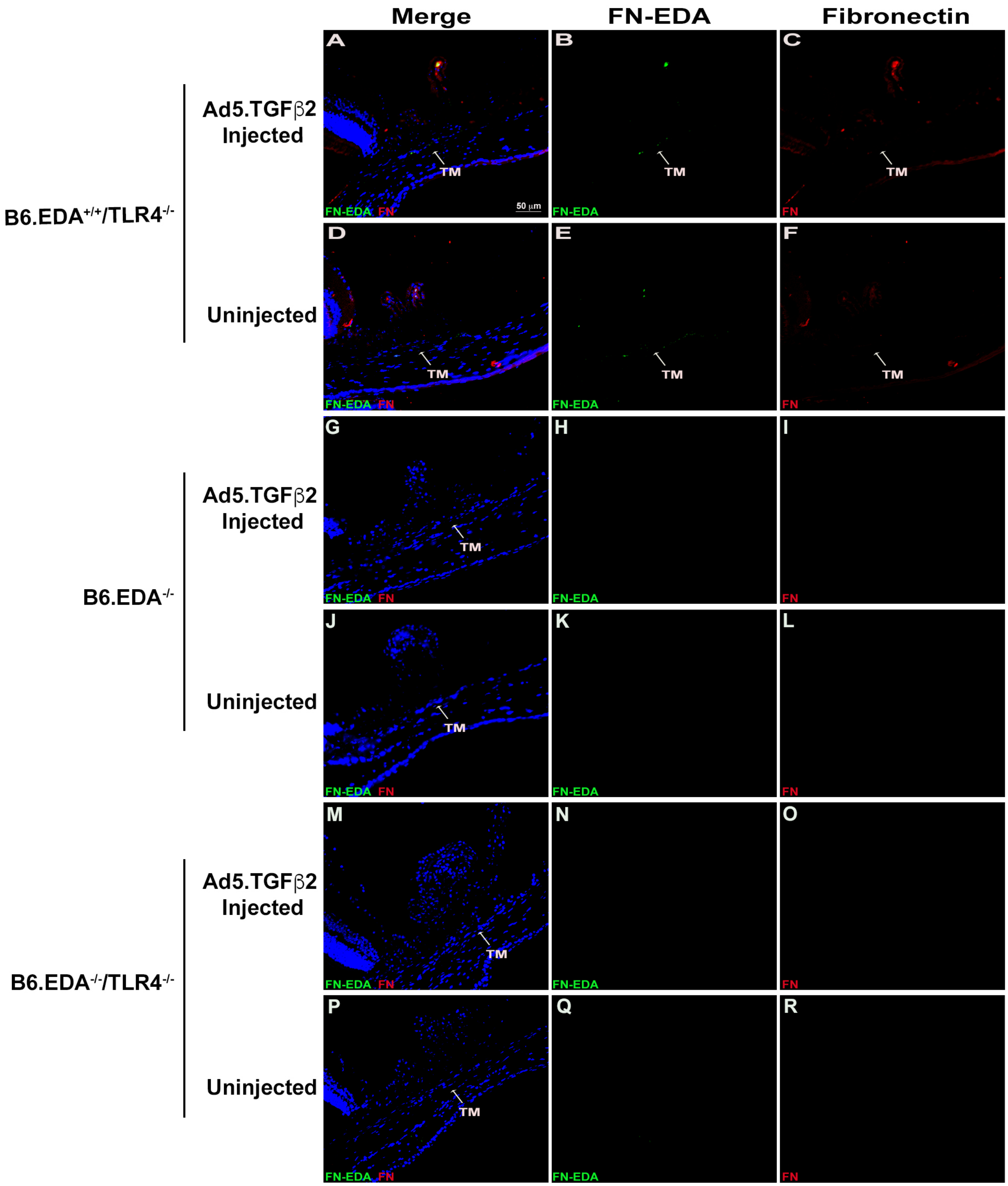

Supplement: Supplementary file 1 — Supplementary Information. [file 41598_2020_66756_MOESM1_ESM.pdf]
